# Knowledge, attitudes, and practices of hygiene among children in schools of Central Kazakhstan: a cross-sectional study

**DOI:** 10.3389/fpubh.2025.1595672

**Published:** 2025-06-03

**Authors:** Ulpan Kuandyk, Alua Omarova, Nailya Delellis

**Affiliations:** ^1^Institute of Life Sciences, Karaganda Medical University, Karaganda, Kazakhstan; ^2^Department of Health Science, Central Michigan University, Mount Pleasant, MI, United States

**Keywords:** schoolchildren, parents, personal hygiene, public hygiene, schools

## Abstract

**Background:**

The issue of good hygiene among school-aged children is crucial for public health and sustainable development. Despite the availability of global studies covering countries with different socio-economic conditions, there is a lack of local data reflecting the specifics of Kazakhstan. Therefore, the aim of this study was to determine the level of knowledge, attitude and practice of schoolchildren regarding personal and public hygiene in schools of Central Kazakhstan.

**Methods:**

School-based cross-sectional study was conducted from September 2024 to December 2024 in six schools of Central Kazakhstan. The simple random sampling technique was used to select the school. A computer-generated sequence of random numbers was used to randomly select students. Subsequently, 382 students aged 11–18 years were selected from a total of 72.179 students. The data were collected through a questionnaire using the online Google forms service. It consisted of socio-demographic data and information on knowledge and practice of personal and public hygiene of students in schools. The data were computerized. A statistical analysis of the data was carried out using SPSS (Statistical Package for Social Sciences). *P* < 0.05 with a 95% confidence interval was considered statistically significant.

**Results:**

The results showed significant gender differences in hygiene knowledge and behavior. More than 90% of students knew about the basic rules of personal hygiene, with girls demonstrating higher knowledge and practices compared to boys (e.g., brushing teeth twice a day: 97% of girls vs. 87.8% of boys, *P* < 0.001). Hand washing before eating was known to 99% of girls and 92.8% of boys (*P* < 0.05). In terms of hygiene practices, girls brushed their teeth twice a day more often (68.7% of girls vs. 57.5% of boys, *P* < 0.05). However, there were differences in hygiene compliance at school due to insufficient infrastructure, such as lack of soap, hot water and poor condition of restrooms.

**Conclusion:**

The study revealed a high level of knowledge about personal hygiene among school students in Karaganda, especially among girls. However, practical compliance with hygiene rules was often limited by infrastructural problems in schools. Strengthening hygiene education programs and improving sanitary conditions in educational institutions is necessary.

## 1 Introduction

Hygiene is a set of practices, activities and knowledge aimed at maintaining health and preventing diseases (1). This concept covers both personal hygiene (e.g., regular hand washing, dental and body care, etc.) and public hygiene (2). Lack of knowledge and skills in personal hygiene, as well as insufficient attention to it, negatively affect the overall development of children (3).

One of the major problems faced by students in schools is infectious diseases. Their main triggers are contaminated water, inadequate sanitation, and poor hygiene practices. Repeated infections often worsen the existing health problems in children, negatively affecting their attendance and performance in school and in some cases can lead to death (4).

Non-attendance of school remains a significant problem among children, with around 75% of all absences due to illness (5). The research suggests that poor hand hygiene among students accounts for 272 million days of absence from school each year worldwide (6), which can lead to poor academic performance and increase the likelihood of expulsion from school (7). A survey in Palestine found that 68% of students had reported washing their hands with soap after using the toilet, playing and eating (8). Meanwhile, a study in India found that although most students had correct knowledge about handwashing before eating, brushing their teeth, rinsing their mouth after eating and combing their hair, this knowledge does not always develop into appropriate practices. This highlights the importance of strategies to sustain behavior change (9, 10).

Many health problems affecting school students can be avoided by actively promoting personal hygiene among both the children and their families (11). Hygiene knowledge is often transmitted to children from parents and schools. This has a direct impact on health and is linked to socio-cultural factors (12). However, knowledge, attitudes and practices of hygiene among students show certain differences depending on their gender (13).

School age is a critical period in a child's overall development. During it, children develop the skills needed to contribute to their peer group (14). If students gain health-promoting skills, they are more likely to maintain these behaviors throughout their lives (15). This may mean that interventions in hygiene practices should be implemented as early as possible, which will increase the impact of changing children's habits (12).

In Central Kazakhstan, the issues of knowledge, attitudes and personal hygiene among students remain relevant. This is directly related to several Sustainable Development Goals (SDGs). In particular, SDG 3 aims to reduce morbidity and improve health and wellbeing, while SDG 4 emphasizes the importance of health education, and SDG 6 stresses the need for schools to provide safe drinking water and sanitation infrastructure (16).

In Central Kazakhstan, several studies have been conducted to examine access to water, sanitation and hygiene in urban and rural areas (17–24). However, there have been no studies assessing the knowledge and practices of personal and public hygiene among schoolchildren in schools of Central Kazakhstan. To fill this gap, this study was conducted in those schools for the first time. The objective of this article was to determine the level of knowledge, attitude and practice of schoolchildren regarding personal and public hygiene in schools of Central Kazakhstan. The results of this study will form the basis for developing future programs to improve hygiene among students in Kazakhstan.

## 2 Materials and methods

### 2.1 Study design and participants

This cross-sectional study was conducted in Karaganda city, Central Kazakhstan. Data were collected from September 2024 to December 2024. To conduct the study, the researchers first obtained official permission from the Karaganda Education Department. Then, they contacted the school principals directly to agree on a schedule for the survey in each of the selected schools.

We collected the cross-sectional data on personal and public hygiene knowledge and practices from students and their parents to assess children's personal and public hygiene skills. In addition, school administrative staff was interviewed to determine accessibility of water supply, sanitation, and hygiene resources in six educational institutions (e.g., availability of clean water, soap, toilets, and other necessary supplies). A total of 382 students aged 11–18 years old (14.69 ± 0.21) studying in grades five to eleven in schools of Karaganda city participated in the study. These age groups were chosen because the reading skills of this age allow students to complete the questionnaire within the allotted time. The inclusion criteria were students aged 11–18 years, available at the time of data collection, and willing to participate in the study. And the exclusion criteria were school-age children out of the mentioned age range.

### 2.2 Sample size and sampling

**Figure 1:**
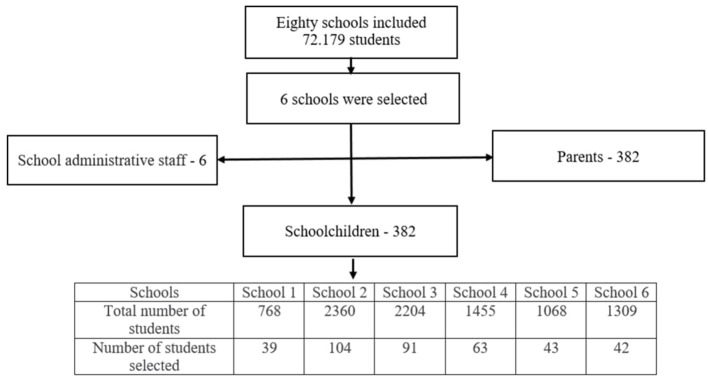
Schematic representation of participants.

For this study, we considered the sample size using OpenEpi software and population proportion formula based on a study conducted in Ethiopia (25). The concentration size N = 72.179, precision (d) = 5%, design effect = 2 and power = 95% CI were taken. A two-stage probability sampling procedure was used to select study participants. There are 80 schools under the supervision of the Karaganda Education Department. In 2022–2023 academic years, 72.179 students were studying in grades 1–11 of the 80 schools mentioned above. In the first stage, six schools were selected out of 80 using a simple random sampling. In the second stage, a random sample of students from grades 5–11 was drawn from each of the selected schools, resulting in 382 respondents being included in the study. A computer-generated sequence of random numbers was used to randomly select students. In addition, a higher number of students were noted from schools with higher total enrollment (Figure 1).

### 2.3 Data collection tools and methods

An online questionnaire with closed structured questions was based on the literature review (26–28). The questionnaire was bilingual (Kazakh and Russian) and consisted of four sections:

I. Demographic data: this section aimed to collect information on the socio-economic status of students and their parents. Its questions covered gender, age, and age group (11–15 years and 16–18 years). For parents, some additional questions determined whether the respondent was a mother or father, their marital status, level of education, family composition, and whether the children had any chronic diseases.
II. Knowledge: A total of 18 questions (eleven of which were yes/no/don't know and seven multiple choice questions with one correct answer) were used to determine the level of students' knowledge about personal and public hygiene in two aspects: (i) Personal Hygiene (seven questions assessing the children's knowledge about personal hygiene including frequency of brushing teeth and replacing toothbrushes, choice of toothbrush hardness, rules of individual use, necessity and methods of cleaning tongue, as well as regularity of washing) and (ii) Hand Hygiene (eight questions to assess children's awareness of hand washing in everyday situations—before meal and cooking, and after meal, going to the toilet, coughing, sneezing or blowing their nose, as well as the use of soap and drying hands after washing; three questions about hand washing in the context of public hygiene: the need to wash hands after contact with garbage or playing with friends, and the use of soap when washing hands at school).
III. Attitude: This section included four questions to identify children's attitudes toward personal hygiene and hand washing. The questions covered the following topics: Can avoiding a shower for a long time lead to infectious skin diseases?, Can a long period of not washing your hair lead to the proliferation of microbes?, Can uncovered abrasions or cuts on your fingers and hands cause cross-infection?, and Can hand washing prevent intestinal infectious and parasitic diseases (dysentery, hepatitis A, ascariasis and giardiasis)?
IV. Practices: A total of 15 questions aimed at three main aspects of hygiene practices: (i) Personal Hygiene (seven questions about personal hygiene practices of children); (ii) Hand Hygiene (eight questions assessing children's awareness of hand hygiene and six questions about handwashing in the context of public hygiene. They covered frequency of handwashing at school, use of soap, hygiene before eating, reasons for possible neglect, hand drying, problems with the condition of restrooms, and compliance with handwashing instructions posted in the restroom); (iii) five questions for parents about how their children perform hand hygiene rules.

### 2.4 Statistical analysis

The data were collected through a questionnaire using the online Google forms service. The collected data were processed using IBM SPSS Statistics © (version 17.0). The mean ± SD was calculated for children's age. Frequencies, percentages and 95% confidence intervals (CI) were calculated for children's gender, age and age groups as well as for parents, their marital status, education, chronic diseases in children and the family wealth index. Pearson's chi-square test and Fisher's exact test (with values of the minimum expected phenomenon < 10) were used to assess the level of hygiene knowledge, attitude and practice among the participants by gender and age groups. A *P* < 0.05 was considered statistically significant.

### 2.5 Ethical considerations

Ethical approval was obtained from the Local Bioethics Commission at Karaganda Medical University, Kazakhstan on 06 February 2024 (assigned No. 9 on 29.12.2023). Written informed consents were obtained from all participants and their parents or guardians, as well as from school principals and people responsible for the child's education. Consent forms included contact details of the senior researcher for possible communication. Both forms described the objective of the study and its process. Participants were informed that their participation was voluntary. In addition, parents were given an explanation of the purpose of the questionnaire before the study began, and school administration asked them to allow their children to participate. They were also informed that they could withdraw their participation at any time without negative consequences.

## 3 Results

### 3.1 Characteristics of participants

Table 1 shows the main characteristics of participants. The study included 382 students (47.4% boys and 52.6% girls). Their mean age ± (SD) was 14.69 ± (0.21) years old with a range from 11 to 18 years. The students were divided into two groups: 11–15 years (51.3%) and 16–18 years (48.7%).

**Table 1:** Socio-demographic profile of participants (*n* = 382).

**Characteristics**		**Frequency (*n* = 382)**	**Percentage (%)**	**95% CI**
Child's gender	Boys	181	47.4%	42.3–52.5
	Girls	201	52.6%	47.5–57.7
Age, Me ± (SD)	11–18 years old, 14.69±(0,21)	382	100%	88.1–100
Age group	11–15	196	51.3%	46.2–56.4
	16–18	186	48.7%	43.6–53.8
Parent category	Mother	355	92.9%	89.9–95.3
	Father	24	6.3%	4.1–9.2
	Grandmother	3	0.8%	0.2–2.3
Family status	Two-parent family	320	83.8%	79.7–87.3
	Single-parent family	57	14.9%	11.5–18.9
	Don't know	5	1.3%	0.4–3.0
Mother's education	Secondary education	103	27%	22.6–31.7
	Higher education (bachelor's degree)	233	61%	55.9–65.9
	Master's degree	34	8.9%	6.2–12.2
	PhD	12	3.1%	1.6–5.4
Father's education	Secondary education	105	27.5%	23.1–32.3
	Higher education (bachelor's degree)	226	59.2%	54.0–64.1
	Master's degree	11	2.9%	1.4–5.1
	PhD	6	1.6%	0.6–3.4
	Don't know	34	8.9%	6.2–12.2
Does your child have any of the following chronic diseases?	Respiratory diseases	44	11.5%	8.5–15.2
	Infectious and parasitic diseases	1	0.3%	0.0–1.4
	Diseases of the digestive system	9	2.4%	1.1–4.4
	Diseases of the endocrine system	4	1%	0.3–2.7
	Diseases of the blood, hematopoietic organs and individual disorders	5	1.3%	0.4–3.0
	Diseases of the musculoskeletal system and connective tissue	14	3.7%	2.0–6.1
	No chronic disease	305	79.8%	75.5–83.8
Family Welfare Index	We easily live on our income for the whole month	149	39%	34.1–44.1
	We manage to live paycheck to paycheck without problems	139	36.4%	31.6–41.4
	We barely manage to live paycheck to paycheck	80	20.9%	17.0–25.4
	We are struggling to make ends meet	14	3.7%	2.0–6.1

Me, mean age; SD, standard deviation; CI, Confidence interval.

A total of 382 parents agreed to participate in the survey; the vast majority of them were mothers (92.9%). Most mothers had higher education (61%), and some of them also hold a master's degree or PhD (12%). Many respondents were married, and their children are raised in two-parent families (83.8%). A significant proportion of children (79.8%) did not have any chronic diseases. The majority of families lived on their income for the entire month without problems (39%) or until they received their next paycheck (36.4%).

### 3.2 Participants' knowledge, attitudes, and practices of personal and public hygiene

Table 2 summarizes the study results about knowledge, attitude and practice of personal hygiene by age groups. Participants of both genders had good indicators of personal hygiene knowledge; however, they were significantly higher among girls. The predominant part of participants of both genders are convinced of the need to brush their teeth twice a day (morning and evening). However, this indicator is significantly higher among girls (97% of girls vs. 87.8% of boys, *P* < 0.001). The majority of students also were of the opinion that a toothbrush should be replaced every 3–4 months (88.6% of girls vs. 72.4% of boys, *P* < 0.001). As for age groups, the majority of students adhere to the opinion that a toothbrush should be replaced every 6 months (11–15 years—62.8% vs. 16–18 years—45.7%, *P* < 0.05). Regarding toothbrush hardness, students mostly realized the need to use soft or medium hardness brushes (88.6% of girls vs. 77.9% of boys, P < 0.05). Almost all participants knew that a toothbrush should be used exclusively individually (100% of girls and 99.4% of boys, *P* = 0.474). In addition, most participants of both genders recognized the need to clean their tongue (89.6% of girls vs. 78.5% of boys, *P* < 0.05). Moreover, girls were more likely to know that a special tongue brush should be used to clean their tongue (73.1% of girls vs. 54.1% of boys, *P* < 0.05). Washing one's face in the morning and evening is the most common practice among both genders (58.2% of boys and 56.9% of girls), with no statistically significant differences between boys and girls (*P* = 0.619).

**Table 2:** Knowledge, attitude and practice of personal hygiene (*n* = 382).

**KAP of personal hygiene**	**Gender**		***P***	**Age group**		***P***
	**Boys**, ***n*** **(%)**	**Girls**, ***n*** **(%)**		**11–15 years old**, ***n*** **(%)**	**16–18 years old**, ***n*** **(%)**	
**Knowledge of personal hygiene**						
How many times a day should you brush your teeth?			0.001^*^			0.591
Once a day (only in the morning)	22 (12.2)	6 (3)		13 (6.6)	15 (8.1)	
Twice a day (in the morning and in the evening)^**^	159 (87.8)	195 (97)		183 (93.4)	171 (91.9)	
How often should you replace your toothbrush?			0.001^*^			0.007^*^
Every 3–4 months^**^	131 (72.4)	178 (88.6)		53 (27.0)	71 (38.2)	
Once every 6 months	18 (9)	24 (13.3)		123 (62.8)	85 (45.7)	
Once a year	2 (1)	9 (5)		3 (1.5)	8 (4.3)	
Don't know	3 (1.5)	17 (9.4)		17 (8.7)	22 (11.8)	
Which toothbrush should I use?			0.024^*^			0.079
Very hard	5 (2.8)	1 (0.5)		5 (2.6)	1 (0.5)	
Hard	16 (8.8)	12 (6)		13 (6.6)	15 (8.1)	
Soft or medium^**^	141 (77.9)	178 (88.6)		167 (85.2)	152 (81.7)	
Very soft	6 (3.3)	6 (3)		7 (3.6)	5 (2.7)	
Don't know	13 (7.2)	4 (2)		4 (2.)	13 (7)	
How can you use your toothbrush?			0.474			1.000
Individually^**^	180 (99.4)	201 (100)		195 (99.5)	186 (100)	
You can lend it to a family member or friend if necessary.	1 (0.6)	0 (0)		1 (0.5)	0 (0)	
Is it necessary to clean your tongue?			0.006^*^			0.475
Yes^**^	142 (78.5)	180 (89.6)		168 (85.7)	154 (82.8)	
No	11 (6.1)	3 (1.5)		5 (2.6)	9 (4.8)	
Don't know	28 (15.5)	18 (9)		23 (11.7)	23 (12.4)	
What should I use to clean my tongue?			0.002^*^			0.338
Toothbrush	51 (28.2)	32 (15.9)		42 (21.4)	41 (22)	
Special tongue brush^**^	98 (54.1)	147 (73.1)		128 (65.3)	117 (62.9)	
Mouthwash	15 (8.3)	14 (7)		13 (6.6)	16 (8.6)	
Dental floss	4 (2.2)	2 (1)		1 (0.5)	5 (2.7)	
Don't know	13 (7.2)	6 (3)		12 (6.1)	7 (3.8)	
How often should you wash your face?			0.619			0.743
Only in the morning	63 (34.8)	66 (32.8)		67 (34.2)	62 (33.3)	
Only in the evening	12 (6.6)	17 (8.5)		16 (8.2)	13 (7)	
In the morning and in the evening^**^	103 (56.9)	117 (58.2)		110 (56.1)	110 (59.1)	
Don't know	3 (1.7)	1 (0.5)		3 (1.5)	1 (0.5)	
**Attitude toward personal hygiene**						
Can avoiding showering for long periods of time lead to skin infections?			0.024^*^			0.001^*^
Yes ^**^	145 (80.1)	176 (87.6)		130 (66.3)	153 (82.3)	
No	12 (6.6)	3 (1.5)		21 (10.7)	7 (3.8)	
Don't know	24 (13.3)	22 (10.9)		45 (23)	26 (14)	
Can a long period of not washing hair lead to the proliferation of microbes?			0.029^*^			0.382
Yes ^**^	156 (86.2)	188 (93.5)		173 (88.3)	171 (91.9)	
No	5 (2.8)	5 (2.5)		7 (3.6)	3 (1.6)	
Don't know	20 (11)	8 (4)		16 (8.2)	12 (6.5)	
**Personal hygiene practices**						
How many times do you brush your teeth?			0.023^*^			0.096
Once a day (morning)	77 (42.5)	63 (31.3)		64 (32.7)	76 (40.9)	
Twice a day (morning and evening)	104 (57.5)	138 (68.7)		132 (67.3)	110 (59.1)	
How long has it been since you used your toothbrush?			0.021^*^			0.007^*^
Three-four months	65 (35.9)	59 (29.4)		53 (27)	71 (38.2)	
Half a year	85 (47)	123 (61.2)		123 (62.8)	85 (45.7)	
A year	8 (4.4)	3 (1.5)		3 (1.5)	8 (4.3)	
Never replaced it	23 (12.7)	16 (8)		17 (8.7)	22 (11.8)	
Which toothbrush do you use to brush your teeth?			0.004^*^			0.112
Very hard	6 (3.3)	3 (1.5)		6 (3.1)	3 (1.6)	
Hard	34 (18.8)	15 (7.5)		18 (9.2)	31 (16.7)	
Soft or medium	132 (72.9)	174 (86.6)		164 (83.7)	142 (76.3)	
Very soft	9 (5)	9 (4.5)		8 (4.1)	10 (5.4)	
How is your toothbrush used?			0.011^*^			1.000
Individually	155 (85.6)	188 (93.5)		195 (99.5)	186 (100)	
You can lend it to a family member or friend if necessary	26 (14.4)	13 (6.5)		1 (0.5)	0 (0)	
Do you clean your tongue?			0.002^*^			0.159
Yes	17 (9.4)	12 (6)		150 (76.5)	133 (71.5)	
No	119 (65.7)	164 (81.6)		29 (14.8)	41 (22)	
Don't know	45 (24.9)	25 (12.4)		17 (8.7)	12 (6.5)	
What do you use to clean your tongue?			0.022^*^			0.218
Toothbrush	64 (35.4)	79 (39.3)		68 (34.7)	75 (40.3)	
Special tongue brush	69 (38.1)	93 (46.3)		92 (46.9)	70 (37.6)	
Mouthwash	21 (11.6)	9 (4.5)		16 (8.2)	14 (7.5)	
Dental floss	2 (1.1)	0 (0)		0 (0.0)	2 (1.1)	
Nothing	25 (13.8)	20 (10)		20 (10.2)	25 (13.4)	
How often do you wash your face?			0.008^*^			0.778
Only in the morning	23 (12.7)	11 (5.5)		15 (7.7)	19 (10.2)	
Only in the evening	29 (16.0)	25 (12.4)		27 (13.8)	27 (14.5)	
In the morning and in the evening	121 (66.9)	162 (80.6)		149 (76)	134 (72)	
I don't wash my face	8 (4.4)	3 (1.5)		5 (2.6)	6 (3.2)	

* The chi-square test is significant at the P < 0.05 level of significance;

** The correct answer.

In addition, Table 2 has two questions about children's attitudes toward personal hygiene, which turned out to be relatively positive. In the question about personal hygiene, more girls compared to boys noted that a long time without showering could lead to infectious skin diseases (87.6% of girls vs. 80.1% of boys, *P* < 0.05). Many students across the age groups held this opinion (11–15 years old—66.3% vs. 16–18 years old—82.3%, *P* < 0.05). A larger proportion of girls also responded that a long period of not washing hair could lead to the proliferation of germs (93.5% of girls vs. 86.2% of boys, *P* < 0.05).

Table 2 also shows the data about hygiene habits of boys and girls, including oral care routines, hand washing, and use of skin care products. Most participants of both genders brushed their teeth twice a day (morning and evening), but this figure was significantly higher among girls (68.7% compared to 57.5% among boys, *P* < 0.05). However, many participants reported that they replaced their toothbrush about 6 months ago; girls did this significantly more often than boys (61.2% of girls vs. 47% of boys, *P* < 0.05). These figures were also similar among the age groups (11–15 years—62.8% vs. 16–18 years—45.7%, *P* < 0.05). As for the hardness of the toothbrushes, students predominantly used soft or medium hardness ones (86.6% of girls vs. 72.9% of boys, *P* < 0.05). Almost all participants used a toothbrush only for personal use (93.5% of girls and 85.6% of boys, *P* < 0.05). Most participants of both genders practiced tongue cleaning, but girls did this significantly more often (81.6% of girls vs. 65.7% of boys, *P* < 0.05). In addition, girls predominantly used special brushes for cleaning the tongue (46.3% of girls vs. 38.1% of boys, *P* < 0.05). Among participants of both genders, morning and evening washing was more common in girls (80.6%) than in boys (66.9%), *P* < 0.05.

Table 3 summarizes the study results about knowledge, attitude and practice of hand hygiene. Most of the responses indicated good knowledge, with more than 80% of the respondents agreeing with the best approaches to hand hygiene. A higher proportion of girls compared to boys knew that hand washing was necessary before eating (99% of girls vs. 92.8% of boys, *P* < 0.05), after eating (89.1% of girls vs. 84% of boys, *P* = 0.242), before preparing food (100% of girls vs. 92.8% of boys, *P* < 0.05), after using the toilet (99.5% of girls vs. 92.8% of boys, *P* < 0.05), after handling garbage (99% of girls vs. 92.3% of boys, *P* < 0.05), and after playing with friends (94% of girls vs. 82.3% of boys, *P* < 0.001). Many participants of both genders responded that soap and water were the best way to wash hands (~98.5, *P* = 0.992). In addition, a higher proportion of girls than boys responded that it was necessary to wash hands with soap both at home (99.5% of girls vs. 92.8% of boys, *P* < 0.05) and at school (91% of girls vs. 81.2% of boys, *P* < 0.05), and to dry hands after washing (88.1% of girls vs. 79.6% of boys, *P* < 0.001). However, the majority of students across the age groups believed that it was necessary to wash their hands with soap at school (11–15 years old—80.6% vs. 16–18 years old—92.5%, *P* < 0.05). The majority (91% of girls and 85.1% of boys, P = 0.088) of children, regardless of gender, responded that it was necessary to wash hands after coughing, sneezing or blowing their nose.

**Table 3:** Knowledge, attitude and practice of hand hygiene (*n* = 382).

**KAP of hand hygiene**	**Gender**		***P***	**Age group**		***P***
	**Boys**, ***n*** **(%)**	**Girls**, ***n*** **(%)**		**11–15 years old**, ***n*** **(%)**	**16–18 years old**, ***n*** **(%)**	
**Hand washing knowledge**						
Is it necessary to wash your hands before eating?			0.008^*^			0.418
Yes^**^	168 (92.8)	199 (99)		188 (95.9)	179 (96.2)	
No	7 (3.9)	1 (0.5)		3 (1.5)	5 (2.7)	
Don't know	6 (3.3)	1 (0.5)		5 (2.6)	2 (1.1)	
Is it necessary to wash your hands after eating?			0.242			0.438
Yes^**^	152 (84)	179 (89.1)		174 (88.8)	157 (84.4)	
No	14 (7.7)	8 (4)		9 (4.6)	13 (7)	
Don't know	15 (8.3)	14 (7)		13 (6.6)	16 (8.6)	
Is it necessary to wash your hands before preparing food?			0.001^*^			0.751
Yes^**^	168 (92.8)	201 (100)		188 (95.9)	181 (97.3)	
No	5 (2.8)	0 (0)		3 (1.5)	2 (1.1)	
Don't know	8 (4.4)	0 (0)		5 (2.6)	3 (1.6)	
Is it necessary to wash your hands after using the toilet?			0.002^*^			0.286
Yes^**^	168 (92.8)	200 (99.5)		187 (95.4)	181 (97.3)	
No	5 (2.8)	1 (0.5)		5 (2.6)	1 (0.5)	
Don't know	8 (4.4)	0 (0)		4 (2)	4 (2.2)	
Is it necessary to wash your hands after handling garbage?			0.005^*^			0.355
Yes^**^	167 (92.3)	199 (99)		185 (94.4)	181 (97.3)	
No	6 (3.3)	1 (0.5)		5 (2.6)	2 (1.1)	
Don't know	8 (4.4)	1 (0.5)		6 (3.1)	3 (1.6)	
Is it necessary to wash your hands after playing with friends?			0.001^*^			0.210
Yes^**^	149 (82.3)	189 (94)		177 (90.3)	161 (86.6)	
No	12 (6.6)	7 (3.5)		6 (3.1)	13 (7)	
Don't know	20 (11)	5 (2.5)		13 (6.6)	12 (6.5)	
What is the best way to wash your hands?			0.992			0.242
With water only	2 (1.0)	2 (1.1)		3 (1.5)	1 (0.5)	
With water and soap^**^	198 (98.5)	178 (98.3)		191 (97.4)	185 (99.5)	
Don't know	1 (0.5)	1 (0.6)		2 (1)	0 (0)	
Is it necessary to wash your hands with soap at home?			0.002^*^			0.511
Yes^**^	168 (92.8)	200 (99.5)		188 (95.9)	180 (96.8)	
No	6 (3.3)	1 (0.5)		3 (1.5)	4 (2.2)	
Don't know	7 (3.9)	0 (0)		5 (2.6)	2 (1.1)	
Is it necessary to wash hands with soap at school?			0.020^*^			0.003^*^
Yes ^**^	147 (81.2)	183 (91)		158 (80.6)	172 (92.5)	
No	16 (8.8)	8 (4)		18 (9.2)	6 (3.2)	
Don't know	18 (9.9)	10 (5)		20 (10.2)	8 (4.3)	
Is it necessary to dry your hands after washing?			0.001^*^			0.594
Yes^**^	144 (79.6)	177 (88.1)		168 (85.7)	153 (82.3)	
No	23 (12.7)	5 (2.5)		12 (6.1)	16 (8.6)	
Don't know	14 (7.7)	19 (9.5)		16 (8.2)	17 (9.1)	
Should you wash your hands after coughing, sneezing or blowing your nose?			0.088			0.751
Yes^**^	154 (85.1)	183 (91)		183 (93.4)	177 (95.2)	
No	11 (6.1)	4 (2)		6 (3.1)	4 (2.2)	
Don't know	16 (8.8)	14 (7)		7 (3.6)	5 (2.7)	
**Attitude toward hand washing**						
Can uncovered abrasions or cuts on fingers and hands cause cross-contamination?			0.029^*^			0.628
Yes ^**^	144 (79.6)	161 (80.1)		158 (80.6)	147 (79)	
No	13 (7.2)	4 (2)		10 (5.1)	7 (3.8)	
Don't know	24 (13.3)	36 (17.9)		28 (14.3)	32 (17.2)	
Does hand washing prevent intestinal infectious and parasitic diseases (dysentery, hepatitis A, ascariasis and giardiasis)?			0.715			0.001^*^
Yes ^**^	131 (72.4)	152 (75.6)		130 (66.3)	153 (82.3)	
No	15 (8.3)	13 (6.5)		21 (10.7)	7 (3.8)	
Don't know	35 (19.3)	36 (17.9)		45 (23)	26 (14)	
**Hand washing practice**						
Where were you taught how to wash your hands properly?			0.811			0.103
At school	15 (8.3)	20 (10)		22 (11.2)	13 (7)	
At home	112 (61.9)	124 (61.7)		128 (65.3)	108 (58.1)	
On social networks	13 (7.2)	13 (6.5)		9 (4.6)	17 (9.1)	
From friends	3 (1.7)	1 (0.5)		2 (1)	2 (1.1)	
Myself	38 (21)	43 (21.4)		35 (17.9)	46 (24.7)	
How do you wash your hands at school?			0.129			0.438
Only with water	86 (47.5)	80 (39.8)		10 (5.1)	13 (7)	
With water and soap	95 (52.5)	121 (60.2)		186 (94.9)	173 (93)	
How do you wash your hands at home?			0.077			0.512
Only with water	15 (8.3)	8 (4)		82 (41.8)	84 (45.2)	
With water and soap	166 (91.7)	193 (96)		114 (58.2)	102 (54.8)	
How many seconds do you wash your hands?			0.479			0.871
40 s or more	94 (51.9)	92 (45.8)		98 (50)	88 (47.3)	
<40 s	67 (37)	85 (42.3)		76 (38.8)	76 (40.9)	
Don't know	20 (11)	24 (11.9)		22 (11.2)	22 (11.8)	
How often do you wash your hands before eating at school?			0.689			0.132
Every time	97 (53.6)	119 (59.2)		119 (60.7)	97 (52.2)	
Often	38 (21)	35 (17.4)		37 (18.9)	36 (19.4)	
Sometimes	19 (10.5)	23 (11.4)		22 (11.2)	20 (10.8)	
Hardly ever	9 (5)	10 (5)		8 (4.1)	11 (5.9)	
Never	18 (9.9)	14 (7)		10 (5.1)	22 (11.8)	
Do you taste and serve food with protected hands?			0.971			0.614
Every time	131 (72.4)	149 (74.1)		139 (70.9)	141 (75.8)	
Often	19 (10.5)	19 (9.5)		21 (10.7)	17 (9.1)	
Sometimes	15 (8.3)	14 (7)		14 (7.1)	15 (8.1)	
Hardly ever	5 (2.8)	7 (3.5)		7 (3.6)	5 (2.7)	
Never	11 (6.1)	12 (6)		15 (7.7)	8 (4.3)	
Do you wash your hands before handling raw food?			0.607			0.951
Every time	148 (81.8)	171 (85.1)		162 (82.7)	157 (84.4)	
Often	18 (9.9)	21 (10.4)		20 (10.2)	19 (10.2)	
Sometimes	10 (5.5)	7 (3.5)		10 (5.1)	7 (3.8)	
Hardly ever	3 (1.7)	1 (0.5)		2 (1)	2 (1.1)	
Never	2 (1.1)	1 (0.5)		2 (1)	1 (0.5)	
How did you usually wash your hands during the last 30 days at school?			0.006^*^			0.371
I haven't washed my hands at school for the last 30 days.	30 (16.9)	25 (12.4)		23 (11.7)	32 (17.5)	
In a bowl or basin of water used by others	19 (10.7)	7 (3.5)		12 (6.1)	14 (7.7)	
In a dish or bowl of water used only by me	22 (12.4)	18 (9)		22 (11.2)	18 (9.8)	
Under running water	107 (60.1)	151 (75.1)		139 (70.9)	119 (65)	
How often do you follow the hand-washing instructions posted in the restroom?			0.501			0.243
Every time	46 (25.4)	47 (23.4)		51 (26.0)	42 (22.6)	
Often	28 (15.5)	43 (21.4)		40 (20.4)	31 (16.7)	
Sometimes	40 (22.1)	49 (24.4)		49 (25.0)	40 (21.5)	
Hardly ever	23 (12.7)	23 (11.4)		22 (11.2)	24 (12.9)	
Never	44 (24.3)	39 (19.4)		34 (17.3)	49 (26.3)	

* The chi-square test is significant at the P < 0.05 level of significance;

** The correct answer.

In addition, Table 3 has two questions about children's attitudes toward hand hygiene, which turned out to be relatively positive. A larger proportion of girls noted that uncovered abrasions or cuts on fingers and hands could cause cross-infection (80.1% of girls vs. 79.6% of boys, *P* < 0.05). Regarding the risk of transmission of intestinal infectious and parasitic diseases (such as dysentery, hepatitis A, ascariasis and giardiasis) through contaminated hands, the majority of children noted that regular hand washing was a remedy (75.6% of girls and 72.4% of boys, *P* = 0.715). Moreover, the majority of participants in these age groups noted the same (11–15 years—66.3% vs. 16–18 years—82.3%, *P* < 0.05).

Table 3 also shows the handwashing practice among the participants of both genders. The overwhelming majority of students received information about proper hand washing from their families: among boys 61.9% and among girls 61.7% (*P* = 0.811). Most of them, predominantly girls, washed their hands using soap and water at school (60.2% of girls vs. 52.5% of boys, *P* = 0.129). However, the majority of the participants washed their hands with soap and water at home (96% of girls vs. 91.7% of boys, *P* = 0.077). Among those who washed their hands for 40 s or more, boys predominated (51.9% of boys vs. 45.8% of girls, *P* = 0.479). Most of the participants washed their hands before eating at school, with girls doing so more often (59.2% of girls vs. 53.6% of boys, *P* = 0.689). In addition, girls were more likely to practice proper hygiene when preparing food, including more frequently tasting or serving food with clean hands (74.1% of girls vs. 72.4% of boys, *P* = 0.971) and washing their hands every time before handling raw food (85.1% of girls vs. 81.8% of boys, *P* = 0.607). When asked how participants usually washed their hands at school over the past 30 days, many reported washing their hands under running water (75.1% of girls vs. 60.1% of boys, *P* < 0.05). Boys were more likely to follow the hand-washing instructions posted in the restroom every time they washed their hands, whereas girls did so only sometimes (25.4% of boys vs. 23.4% of girls, *P* = 0.501).

Table 4 summarizes the data on the generally accepted practice of personal hygiene in children under the parents' supervision (*n* = 382). The results show that various aspects of children's hygiene behavior differ by gender, including the cycle of reminders to brush their teeth, the use of soap when washing hands before meals and after using the toilet, as well as the basic hygiene principles. It is noted that boys were reminded to brush their teeth more often than girls (49.2% of boys vs. 37.8% of girls, *P* < 0.05). This is applicable to the parental approach to hygiene education depending on the child's gender. Almost all children washed their hands before eating (94.5% of boys vs. 92.5% of girls, *P* < 0.001). The percentage of children washing their hands after using the toilet was higher among girls (86.2% of boys and 88.1% of girls, *P* = 0.482). Most parents did not know the reasons why their children did not wash their hands with soap at school (52.5% of girls and 48.8% of boys, *P* = 0.088), but many of them provided flexible reasons indicating the lack of soap and other detergents (35.3% of boys and 25.4% of girls, *P* = 0.088). Most parents noted that their children washed hands on their own, without reminders (92.3% of boys and 88.6% of girls, *P* = 0.577).

**Table 4:** Children's practice of personal hygiene under parents' supervision (*n* = 382).

**Indicator**	**Gender**		***P***	**Age groups**		***P***
	**Boys**, ***n*** **(%)**	**Girls**, ***n*** **(%)**		**11–15 years old**, ***n*** **(%)**	**16–18 years old**, ***n*** **(%)**	
Do you remind your child to brush their teeth?			0.025^*^			0.490
Yes	89 (49.2)	76 (37.8)		88 (44.9)	77 (41.4)	
No	92 (50.8)	125 (62.2)		108 (55.1)	109 (58.6)	
Does your child wash his hands with soap before eating?			0.001^*^			0.538
Yes	171 (94.5)	186 (92.5)		186 (94.9)	171 (91.9)	
Hardly ever	9 (5)	11 (5.5)		8 (4.1)	12 (6.5)	
No	0 (0)	1 (0.5)		0 (0.0)	1 (0.5)	
Don't know	1 (0.6)	3 (1.5)		2 (1.0)	2 (1.1)	
Does your child wash his/her hands with soap after using the toilet at home?			0.482			0.635
Yes	156 (86.2)	177 (88.1)		169 (86.2)	164 (88.2)	
Hardly ever	23 (12.7)	19 (9.5)		22 (11.2)	20 (10.8)	
No	1 (0.6)	1 (0.5)		1 (0.5)	1 (0.5)	
Don't know	1 (0.6)	4 (2)		4 (2.0)	1 (0.5)	
If your child does not wash his/her hands with soap at school, why?			0.088			0.336
No time	11 (5.5)	15 (8.3)		14 (7.1)	12 (6.5)	
Lack of desire or skill	3 (1.5)	9 (5.0)		6 (3.1)	6 (3.2)	
No soap or other detergents	71 (35.3)	46 (25.4)		69 (35.2)	48 (25.8)	
No water or limited access to water	18 (9.0)	16 (8.8)		15 (7.7)	19 (10.2)	
Don't know	98 (48.8)	95 (52.5)		92 (46.9)	101 (54.3)	
How does your child wash his/her hands after coming home?			0.577			0.731
On his/her own, without being reminded	167 (92.3)	178 (88.6)		174 (88.8)	171 (91.9)	
On his/her own, after a reminder	10 (5.5)	16 (8.0)		16 (8.2)	10 (5.4)	
On his/her own but after a reminder and under supervision	2 (1.1)	2 (1.0)		2 (1)	2 (1.1)	
On his/her own, but quickly and poorly	2 (1.1)	5 (2.5)		4 (2)	3 (1.6)	

* The chi-square test is significant at the *P* < 0.05 level of significance.

**Figure 2:**
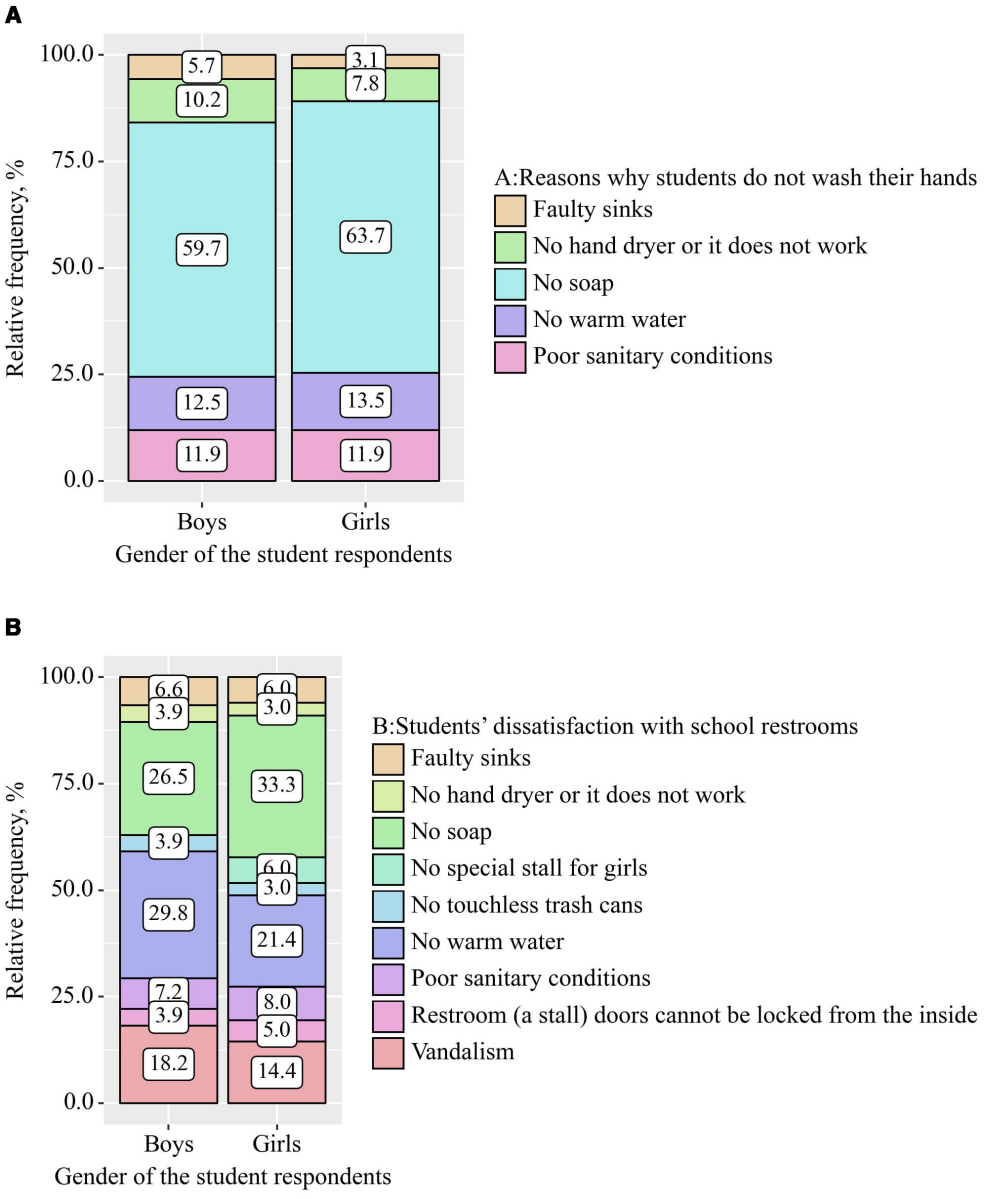
Percentage distribution of reasons why students do not wash their hands and are not satisfied with school restrooms. **(A)** Reasons why students do not wash their hands; **(B)** Students' dissatisfaction with school restrooms.

Figures 2A, B show the distribution diagrams on the reasons why children cannot wash their hands in school restrooms and their dissatisfaction with the conditions there, depending on gender. The data are presented in the form of 100% bar graphs, divided into several color-coded categories. The most significant problem was the lack of soap. This indicator was the dominant reason for both groups, influencing both the reluctance of children to wash their hands during their stay at school (59.7% for boys and 63.7% for girls) and their dissatisfaction with the conditions in school restrooms (26.5% for boys and 33.3% for girls 26.5%). It indicates the systematic nature of this problem in the school infrastructure. An additional complication is the lack of warm water (12.5% for boys vs. 13.5% for girls and 29.8% for boys vs. 21.5% for girls, respectively) and poor sanitation, which showed approximately the same values for boys and girls (~12% and ~7.6%, respectively). These indicators showed an equal distribution of the above problems among gender groups. Another reason for dissatisfaction of children with school restrooms was vandalism (18.2% for boys and 14.4% for girls).

Table 5 presents the data from school administrative staff to determine the level of availability of hygiene infrastructure in six school restrooms (*n* = 6), covering lighting, ventilation, availability of electric heating, toilet paper or hygienic shower, water, soap, hand dryers or disposable paper towels. All six schools always have lighting, ventilation, electric heating, water, and soap. Toilet paper or hygienic shower is always available in three schools (50%, 95% CI: 11.8–88.2), but is completely unavailable in the remaining three (50%, 95% CI: 11.8–88.2). Hand dryers or disposable paper towels are always available in four schools (66.7%, 95% CI: 22.3–95.7), while they are rarely available in two others (33.3%, 95% CI: 4.3–77.7). The provision of lighting, water and electric heating meets standards in all the schools. However, there are differences in the availability of ventilation, toilet paper, soap and hand dryers, indicating the need for improvement of facilities and resources in some institutions.

**Table 5:** Hygienic infrastructure in school restrooms (*n* = 6).

**Indicators**	**Categories**	***n* (%)**	**95% CI**
Lightening	Yes, always	6 (100)	54.1–100.0
Ventilation	Yes, always	5 (83.3)	35.9–99.6
	Yes, in most cases	1 (16.7)	0.4–64.1
Electric heating in the cold season	Yes, always	6 (100)	54.1–100.0
Toilet paper or hygienic shower	Yes, always	3 (50)	11.8–88.2
	No, never	3 (50)	11.8–88.2
Water	Yes, always	6 (100)	54.1–100.0
Soap	Yes, always	4 (66.7)	22.3–95.7
	Yes, in most cases	2 (33.3)	4.3–77.7
Hand dryers or disposable paper towels	Yes, always	4 (66.7)	22.3–95.7
	Hardly ever	2 (33.3)	4.3–77.7

CI, Confidence interval.

Table 6 presents data on the availability and equipment of school restrooms. Special stalls for girls equipped with a bidet or hygienic shower are available in four out of six schools (66.7%, 95% CI: 22.3–95.7), and not available in two others (33.3%, 95% CI: 4.3–77.7). Touchless trash cans (with a foot pedal or sensor) are available in only half of the surveyed schools (50%, 95% CI: 11.8–88.2), indicating the need to improve infrastructure to ensure hygiene. Thus, basic hygiene standards are met in all schools, but there is room for further improvement, especially in ensuring the availability of special restroom stalls for girls and touchless waste disposal facilities.

**Table 6:** School restroom equipment (*n* = 6).

**Questions**	**Categories**	***n* (%)**	**95% CI**
Are there separate restrooms for boys and girls?	Yes	6 (100)	54.1–100.0
Are there restrooms on each floor?	Yes	6 (100)	54.1–100.0
Are the restrooms equipped with closed stalls?	Yes	6 (100)	54.1–100.0
Can the restroom door (or a stall) be locked from the inside?	Yes	6 (100)	54.1–100.0
Are there special stalls for girls (with bidet and/or hygienic shower)?	No	2 (33.3)	4.3–77.7
	Yes	4 (66.7)	22.3–95.7
Are there special stalls for children with disabilities?	Yes	6 (100)	54.1–100.0
Are there any touchless trash cans (with a foot pedal or a touch sensor)?	No	3 (50)	11.8–88.2
	Yes	3 (50)	11.8–88.2

CI, Confidence interval.

## 4 Discussion

This study revealed a stable relationship between the level of knowledge and hygiene behavior among school students in Karaganda city. It is consistent with the results obtained in similar studies in Ethiopia and Saudi Arabia (11, 29). This pattern confirms that student awareness is one of the key factors to form sustainable hygiene habits.

One of the main findings of the study is the presence of pronounced gender differences in hygiene knowledge and behavioral practices. Girls, as a rule, demonstrate a more responsible attitude to personal and public hygiene. Similar gender differences were recorded in the study of Alshammari et al., (30) and a cross-sectional study in Hong Kong (31), where girls showed a higher level of both theoretical knowledge and practical skills in hygiene. This can be explained by the influence of socio-cultural factors and greater sensitivity of girls to their appearance and health.

In terms of hand hygiene knowledge, the proportion of students aware of the need to wash their hands after using the toilet and before meal was higher than in a study in India, where only 21% of respondents were aware of the importance of regular hand washing (32).

Despite adequate knowledge, hygiene practices among children vary significantly depending on the school environment. Our study showed that students were less likely to practice hygiene at school compared to their home. One of the key factors hindering personal hygiene is inadequate infrastructure in educational institutions. Most schools lack soap, hot water, proper ventilation and toilet paper. Even with motivation and knowledge, the lack of basic sanitation makes hygiene practices difficult to implement. These observations point to a critical shortage of hygiene resources in Karaganda schools, a problem that has also been documented in a number of international studies (33–38). This situation highlights the need to review approaches to school hygiene infrastructure and policy. Formation of sustainable hygiene habits is impossible without appropriate facilities and resources. This requires both adequate funding and control over the sanitary condition of educational institutions.

From a government perspective, these results highlight the need to strengthen monitoring of sanitation standards in schools and to include hygiene issues in school monitoring programs. Subsidizing schools for the purchase of hygiene products and the modernization of sanitary facilities should be considered. It is also necessary to include provisions on financing the purchase of hygiene products and the mandatory availability of sanitary and hygienic materials.

In pedagogical practice, the identified differences in knowledge and habits between girls and boys indicate the importance of regular educational activities on personal hygiene, starting from primary school. Hygiene education programs should take into account the gender of students and be aimed at developing sustainable skills through health lessons and integration into other academic subjects.

In the post-COVID reality, when sanitary and hygienic standards for educational institutions have become much stricter, this problem is becoming especially relevant. Providing basic conditions for maintaining hygiene should be a priority in the education system, since it directly affects not only student health, but also the formation of sustainable behavioral models.

The COVID-19 pandemic has demonstrated how vital basic skills of personal hygiene (especially regular and proper handwashing) are to prevent the spread of infectious diseases. However, despite the high level of basic awareness, the results show that there are gender and age differences in the level of practical compliance with hygiene standards. These data highlight the need to revise approaches to health education, focusing on the behavioral component, especially among boys.

The uniqueness of this study also lies in the multi-faceted approach to data collection: not only were the students interviewed, but also their parents and school administration, which ensured a comprehensive analysis of the hygienic environment and behavioral patterns.

Thus, this study expands the existing pool of knowledge about school hygiene and can serve as a basis for the development of local health education programs, especially in the context of strengthening the resilience of the school environment to future epidemiological threats. The development and implementation of these programs in Karaganda schools will help to build the capacity of students' knowledge, attitudes and practices of proper hygiene and focus the attention of policy makers, supervisory authorities and administration on the serious impact of the unsatisfactory school infrastructure on the quality of education and student health.

## 5 Conclusions

In conclusion, it should be noted that the level of hygiene knowledge of students in Karaganda city is closely related to their behavioral practices. Significant gender differences have been revealed: girls demonstrate a higher level of knowledge and compliance with hygiene standards. Despite good awareness, hygiene compliance at school is significantly lower than at home, mainly due to insufficient sanitation infrastructure. The results of the study complement knowledge about school hygiene and can become the basis for local health education programs. They will improve the hygiene skills of students and draw the attention of authorities to the importance of sanitation infrastructure for the student health and quality of education.

## Data Availability

The original contributions presented in the study are included in the article/supplementary material, further inquiries can be directed to the corresponding author.
